# Review of scoliosis-specific exercise methods used to correct adolescent idiopathic scoliosis

**DOI:** 10.1186/s40945-019-0060-9

**Published:** 2019-08-23

**Authors:** Joseph M. Day, Jeremy Fletcher, Mackenzie Coghlan, Terrence Ravine

**Affiliations:** 1 Department of Physical Therapy, School of Education and Health Sciences, University of Dayton, 300 College Park Drive, Dayton, OH 45469-2925 USA; 2 Department of Physical Therapy, University of South Alabama, Mobile, AL USA; 3 University of South Alabama, Mobile, AL USA; 4 Department of Biomedical Sciences, University of South Alabama, Mobile, AL USA

**Keywords:** Scoliosis-specific exercise, Adolescent idiopathic scoliosis, Cobb angles

## Abstract

**Background:**

Adolescent idiopathic scoliosis (AIS) refers to a spinal curvature of an unknown origin diagnosed in otherwise healthy children. A conservative treatment approach includes physiotherapy scoliosis-specific exercises (PSSE) with or without corrective bracing in preventing further spinal column deviation. However, several PSSE types have been developed to facilitate a positive patient outcome and/or preclude surgical remediation. Based on other reviews, there has been insufficient evidence published on the efficacy of PSSEs. In addition, the superiority of PSSE over no intervention or compared to other exercise modes has yet to be determined.

**Methods:**

A comprehensive search of AIS literature, inception through February 2018, was conducted to reveal relevant PSSE articles. Only studies using commonly reported PSSEs were included. Examined databases included PubMed, Scopus, CINAHL Complete, and Physiotherapy Evidence Database (PEDro). Google Scholar search engine was also examined. Article types included randomized or clinical control trials. All articles were published in English or were of English translation. Search parameters were collectively defined by the reviewers and subsequently used to determine included studies. Individual PSSE study methodology quality was determined by the PEDro scale. Effect sizes (Hedge’s g) and their 95% confidence intervals were calculated for Cobb angle between group changes.

**Results:**

Of the initial 24 articles recovered only eight (33%) met the established search criteria. Patient ages from these sources ranged from 11.4–16.2 including both males and females. Examined papers included two Schroth method and six specifying the Scientific Exercise Approach to Scoliosis (SEAS) method. All articles demonstrated positive between group effect sizes for PSSEs. There were no studies that compared one PSSE to another. Determined PEDro scores indicated an overall moderate quality of these studies.

**Conclusions:**

There is insufficient evidence to suggest that both Schroth and SEAS methods can effectively improve Cobb angles in patients with AIS compared to no intervention. There is limited evidence that the SEAS method is more effective at reducing Cobb angles compared to traditional exercises in treating AIS. Overall, this review revealed a noticeable lack of contemporary studies that could be used in answering our questions. Evidence-based medicine (EBM) supplies clinicians with verifiable results from well-designed and managed research studies. Consequently, more and varied studies of higher quality are needed before any definitive determination can be made as to the effectiveness of any PSSE let alone the one offering better patient outcomes.

## Background

Scoliosis is defined as a lateral curvature found either in the lower lumbar, middle thoracic, and/or upper cervical spinal column regions. Curvatures are generally described as either conforming to an “S” or “C” shape [1]. Spinal deformities can result in pain when performing relatively simple tasks such as standing, walking, or lifting objects and is often accompanied by a decreased range of motion. Uncontrolled disease progression may further result in severe pain, to the point of immobility, or even osteoporosis. Adolescent idiopathic scoliosis (AIS) is the usual diagnosis made in healthy children with a spinal curvature of unknown cause of least 10 degrees but less than 50 degrees. It represents the most common scoliosis type affecting 1-3% of adolescents in the United States [2]. Worldwide prevalence of AIS has been more difficult to estimate. Factors such as “varying definitions of scoliosis, study protocols, and age-groups, missing standards for comparison and inclusion of curves <10°” have impacted a true estimate. However, several studies examined by Konieczny et. al. [3] indicated a prevalence of 0.47–5.2 % for AIS.Scoliosis treatment can be separated into either conservative or surgical methods, being based upon patient age, curvature size, and the risk of disease progression. The primary aim of scoliosis management is to stop curvature progression. Non-conservative treatment usually involves surgery to correct spinal deformities but these procedures are not without risk*.* Conservative therapies such as physiotherapy scoliosis-specific exercise (PSSE), with or without concurrent external bracing, are used as an alternative for patients presenting less than a 50-degree curvature. Due to the lack of higher quality studies, systematic reviews in 2014 and 2016 concluded that there was insufficient evidence to make a judgement as to whether conservative treatments were effective in managing this population [4, 5]. Anecdotally, the American Academy of Orthopedic Surgeons agree that PSSEs are often considered to produce just as successful of a patient outcome as does surgery [6].

There are a number of specific types of PSSEs referenced in the literature, but some techniques appear to be prescribed more often than others [7–10]. The four exercise approaches initially considered in this review included the Schroth method, the Scientific Exercise Approach to Scoliosis (SEAS), the Dobosiewicz technique, and the Side-shift program.

The Schroth method was developed by Katharina Schroth in Germany in 1921. This particular method uses a physiotherapeutic approach in strengthening and lengthening any uneven muscle groups. Treatments consists largely of a combination of scoliotic posture correction along with a modification of a patient’s breathing pattern through mirror self-monitoring [11, 12]. Schroth breathing techniques are described as a “rotational breathing”, which aims to lengthen the trunk and correct spinal imbalances [11]. The primary goal is to improve both the patient’s posture and spine alignment mediated by a clinician maintaining proper positioning and utilizing exercise repetition. Using a mirror, the patient is taught to visualize his/her collapsed area(s) needing to be lengthened or contracted. Over time, exercise promotes spinal muscle correction to help stabilize curve(s), mobilize stiff body regions, correct postural alignment, and increase muscle strength/endurance. This process is largely accomplished through axial lengthening, asymmetric sagittal straightening, rotational breathing, and developing frontal sagittal straightening and muscle activation [2].

Based upon the Lyon methodology, the Scientific Exercise Approach to Scoliosis (SEAS) began in the 1960s [13]. SEAS has been described as a “scoliosis-specific active self-correction technique performed without any external aids and incorporated into functional exercises” [14]. The primary goals of SEAS include enhanced posture control, posture rehabilitation, muscle endurance, spinal stability, self-correction, and development of balance stability [15]. Treatment sessions are conducted at least twice a week for 40 minutes each. Unlike the Schroth method these treatments are mostly performed at home. Additionally, SEAS utilizes a teamwork approach involving both clinicians (physician, physiotherapist, orthotist) and family members in generating successful patient outcomes [14].

The Dobosiewicz method, or DoboMed, was established in 1979. It has been described as a “3D auto-correction” technique. This particular technique utilizes a combination of instructional elements including mirrors, photographs, and video all to promote the correct execution of treatment exercises. There are three main objectives. First, a symmetrically positioned pelvis and shoulder girdle. Second, a primary curve mobilization towards a normal posture with a special emphasis on kyphotization or backward displacement of the thoracic spine along with a “lordotization” of the lumbar spine, as required [14]. Third, to achieve stabilization of the corrected spinal position and make it a postural habit of the patient. The DoboMed can be used by itself, in conjunction with bracing, or even prior to surgical correction [7, 16, 17].Mehta first reported on the Side-shift exercise program in 1985, a year after its development. This method involves active correction of the spinal curve through frequent lateral shifting of the trunk relative to the concavity of the curve. The primary objective of the program is to effectively reduce AIS patient spinal deviation by gradually correcting it towards the body midline. The Side Shift method uses similar breathing techniques to the Schroth and DoboMed methods [14]. Exercises are independently performed, which means that patients must be old enough to understand how to properly accomplish prescribed exercises. It may hold its greatest promise as an additional treatment for AIS patients demonstrating an initial Cobb angle between 20°- 32°. However, it has also been suggested that the Side-shift method should only be considered as a secondary treatment method for AIS [15].

These four PSSE methods have shown some promise for improving outcomes in patients with AIS [9, 18, 20, 21, 24]. To the author’s knowledge, three systematic reviews have exclusively investigated the effects of PSSE’s on individuals with AIS and these were published in 2008, 2011, 2013 [9, 18, 19]. Based on these reviews, recommendations were made in favor of PSSE’s for reducing scoliosis curve progression (Cobb angles) in patients with AIS, but several studies were noted to have weak methodological rigor and the heterogeneity of the studies did not allow the author’s to perform additional quantitative analyses, like effect size calculations. The authors of these reviews recommended a continuation of clinical trials with similar outcome measures and full data sets so that comparisons of PSSEs to no interventions and other types of exercises can be made. To that end, the authors have noted that updates to the literature have been made since 2013.Therefore, the purposes of the current review is to 1) determine if there is quantitative evidence that common PSSE’s (Schroth, SEAS, DoboMed, Side-shift methods) are effective at improving Cobb angles in patients with AIS compared to no treatment, 2). determine if there is quantitative evidence that common PSSE’s are effective at improving Cobb angles in patients with AIS compared to standard exercise prescription and if possible, 3) to explore if one PSSE method is more effective at improving Cobb angles compared to other PSSEs in patients with AIS. The author’s hypothesize that all PSSEs will demonstrate objective improvement in Cobb angles in patients with AIS, and based on clinical observation, the Schroth method provides superior results compared to the other methods.

## Methods

### Information sources

In this review, published clinical trials that investigated common PSSEs as a means of conservative treatment interventions for adolescent idiopathic scoliosis were included. PubMed, Scopus, Pedro, Google Scholar (search engine), CINAHL Complete, and Physiotherapy Evidence Database (PEDro) were searched from inception through February 2018 using a predefined search strategy. The following key words were searched in the databases: “idiopathic”; “adolescent”; “scoliosis”; “Schroth method”; “exercise”; “conservative;” “SEAS;” “side-shift;” “Dobosiewicz;” “specific exercises;” “SRS;” “physiotherapy” and various combinations of these terms. Searches were limited to article written in English.

### Eligibility criteria

Inclusion of studies was restricted to the following PICO items. The length of patient follow up was not considered in the criteria.

- *P (population)* individuals diagnosed with adolescent idiopathic scoliosis
- *I (intervention)* utilized some form of one of the aforementioned PSSEs (Schroth, SEAS, DoboMed, Side-shift methods)
- *C (comparison)* control group defined as no treatment, placebo, standard of care or other conservative interventions such as bracing, other PSEEs, or other non-specific exercise intervention
- *O (outcome)* Cobb angles. Studies for which the author was not able to obtain baseline and ending Cobb angles and their associated standard deviations were excluded from our quantitative analysis. For articles that did not report the full data set, the author attempted to obtain the information from the corresponding author via email.

Additionally, it was required that each study be a clinical controlled trial (CCT) or randomized controlled trial (RCT). Studies reporting outcomes on spinal surgery, alternative and integrative medicine, bracing without exercise intervention, or pharmacological interventions were excluded.

### Assessment of methodological quality

Included studies were evaluated using the PEDro scale. Methodological quality was assessed using the following modified rubric based on original cut off score proposed by Maher et al.: 0–4 was considered weak, 5–7 was considered moderate, and 8 or greater was considered to be strong [22]. Assessment of methodological quality was performed independently by two investigators. The two investigators then came to a consensus on any discrepancy in scores.

### Data synthesis

Post intervention effect sizes (bias corrected Hedges’ g) and their 95% confidence intervals were calculated for the mean differences in Cobb angles between the experimental and comparison groups. Mean pre and post-intervention Cobb angles with standard deviations were extracted for the experimental and comparison groups. The extracted data from each available study was analyzed with MetaEasy v1.0.5 – University of Manchester. Effect sizes were interpreted using the following rubric: 0–0.2 was considered as small, 0.21–0.7 was considered as moderate, and > 0.7 was considered as strong [23]. Pooled effect sizes (fixed and random) and measures of heterogeneity (Cochrane Q and I^2^) were calculated using MetaEasy for the studies included in both of the aforementioned questions.

## Results

**Fig. 1:**
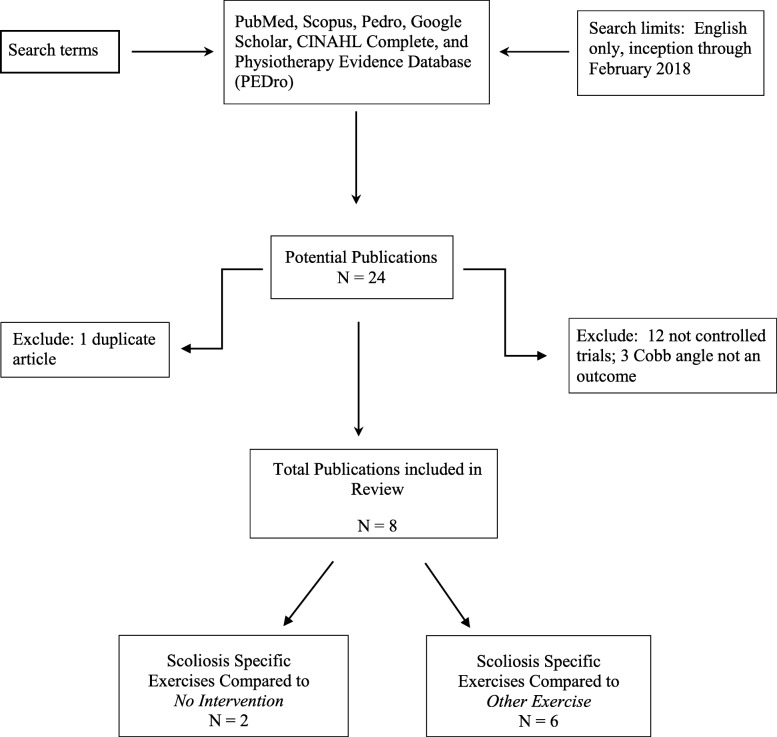
Search Diagram

A total of 24 PSSE articles met the initial search criteria (Appendix). Twelve articles were excluded for not meeting the criteria of a controlled clinical trial. Three additional articles were excluded for not including Cobb angles as an outcome measure. One article was excluded for duplicate information (Fig. 1). Therefore, a total of eight articles were included for this review (Table 1).

**Table 1:** Included scoliosis-specific exercise studies

Article	Exercise method	Patient number	Average patient age	Outcomes measured	Comparison group	Treatment length (days)
Schreiber et al. (2016) [26]	Schroth method	25	13.5	Cobb angles, ATR, SRS-22	Standard of care (*n* = 25)	180
Kuru et al. (2015)	Schroth method	15	13.0	Cobb angles, ATR, SRS-23	HEP (*n* = 15), no treatment (*n* = 15)	42
Zaina et al. (2009) [30]	SEAS	14	14 ± 1.0	Cobb angles, ATR	Supervised exercise group (*n* = 29)	980
Negrini et al. (2008) [31]	SEAS	35	14.3 ± 1.9	Cobb angles, ATR, # of braced patients	Standard of care (*n* = 39)	Not reported
Negrini et al. (2006; page 519) [29]	SEAS 2.0	40	13.3 ± 2.1	Cobb angles, ATR	Supervised exercise group (*n* = 70)	147
Negrini et al. (2006; page 523) [29]	SEAS 2.0	23	12.7 ± 2.2	Cobb angles, ATR	Supervised exercise group (*n* = 25)	364
Monticone et al. (2014) [16]	SEAS (self-correction)	52	12.5 ± 1.1	Cobb angles, ATR, SRS-22	Supervised exercise group (*n* = 51)	1638
Noh et al. (2014) [19]	SEAS (self-correction)	16	13.2	Cobb angles, SRS-22	Supervised exercise group (*n* = 16)	119

*Abbreviations: HEP* home exercise program, *ATR* axial trunk rotation, *SEAS* Scientific Exercise Approach to Scoliosis, *SRS* Scoliosis Research Society Questionnaire, *VC* vital capacity

Of the eight studies, two answered our first questions, is there quantitative evidence that common PSSE’s are effective at improving Cobb angles in patients with AIS when compared to no intervention? In both studies [11, 26] the Schroth method was the experimental group and the control group received observation only. However, in the Schreiber et al. paper a few of the control clients additionally received bracing.

No articles compared one PSSE method to another. Six articles, all SEAS experimental interventions, were compared to a traditional exercises. Despite attempts to contact the corresponding authors, two of the six aforementioned articles had insufficient data to calculate effect sizes [9, 28]. See Table 2 for a qualitative summary of those articles. One Schroth intervention study used an HEP as a control [11]. (This article was also used to answer the first question as there was a Schroth comparison both to an HEP group and observational group) Therefore, a total of seven articles were assessed to answer our second question, does one PSSE method provide superior results compared to standard exercise in patients with AIS?

**Table 2:** Qualitative analysis of studies with insufficient data sets

Author and year	Study design	Interventions and dosages	Results	Qualitative discussion
Negrini et al. (2006b) [29]	CCT	Experimental: Scientific Exercises Approach to Scoliosis 2002 Version (1.5 h session every 2–3 months, 2 40 min sessions independent sessions at a local facility per week; 5 min independent exercise daily) Control: exercise per treating therapist (2–3 times a week for 45–90 min treatment sessions)	Cobb angles improved post intervention only in the experimental group (*p* < .05). The difference in the number of patients requiring bracing was not statistically significant between groups	It should be noted that the quantity of time the therapist spent with the experimental group was reported to be substantially less to the control group. The results of this study should be taken cautiously as much of the details of the methods are left out of the report. Therefore, there is a rather large risk of bias in the study methods.
Negrini et al. (2008) [31]	CCT	Experimental: Scientific Exercises Approach to Scoliosis 2002 Version (1.5 h session every 2–3 months, 2 40 min sessions independent sessions at a local facility per week; 5 min independent exercise daily) Control: exercise per treating therapist (2–3 times a week for 45–90 min treatment sessions)	23.5% of patients in the SEAS group improved while 11.8% worsened in terms of Cobb angles (*p* < .05). 11.1% of patients in the control group improved while 13.9% worsened.	The significant changes in Cobb angles for the intervention group can not be considered clinically significant as they likely did not exceed measurement error.

Of the eight included studies, four (50%) [28–31] included bracing as part of therapy. One study [31] did not specify which type of thoracolumbar-sacral orthosis (TLSO) bracing was prescribed. A separate set of studies used Milwaukee, Boston, and Lyon braces for a total of 5 months [28, 29]. A study by Zaina et al. [30] described brace weaning of 68 patients wearing various brace types including TLSO and cervico-thoraco-lumbo-sacral (CTLSO).

Determined PEDro scores indicated an overall moderate quality of study methods (Table 3) with an average score of 5.9 (range 3–9). The 6 SEAS studies averaged 5 (range 3–9) while the average PEDro score for the 2 Schroth studies was 7.5 (range 7–8).

**Table 3:** Methodological quality of included scoliosis-specific exercise studies

Studies	Score	Eligibility	Random allocation	Concealed allocation	Baseline measure	Blind subjects	Blind therapist	Blind assessor	Adequate follow up	Intention to treat	Between group comparisons	Point Estimate of Variability
Monticone et al	9	Yes	Yes	Yes	Yes	Yes	No	Yes	Yes	Yes	Yes	Yes
Negrini 2006a [28]	3	No	No	No	Yes	No	No	No	No	No	Yes	Yes
Negrini 2006b [29]	3	No	No	No	No	No	No	No	No	No	Yes	Yes
Negrini 2008 [9]	3	Yes	No	Yes	No	No	No	No	Yes	No	Yes	No
Noh et al	7	Yes	Yes	No	Yes	No	No	Yes	Yes	Yes	Yes	Yes
Kuru 2015	7	Yes	Yes	Yes	Yes	No	No	No	Yes	Yes	Yes	Yes
Zaina et al	5	Yes	No	No	Yes	No	No	No	Yes	Yes	Yes	Yes
Schreiber 2016 [26]	8	Yes	Yes	Yes	Yes	No	No	Yes	Yes	Yes	Yes	Yes

### Is there quantitative evidence to suggest that PSSE’s are effective at improving cobb angles in patients with AIS when compared to a control group?

**Fig. 2:**
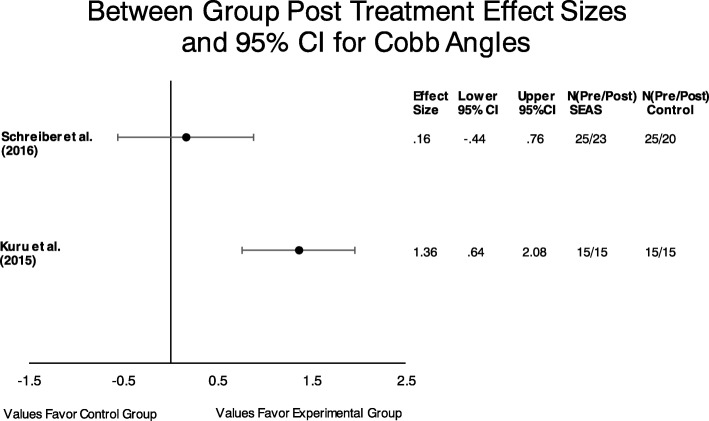
Between group effect sizes were calculated to answer the following question: Is there quantitative evidence to suggest that PSSE’s are effective at improving Cobb angles in patients with AIS when compared to no intervention? Both studies included a Schroth experimental group

Two between group effect sizes and their 95% confidence interval were calculated to answer the question (Fig. 2). Both studies exhibited a positive effect size for reduction in Cobb angles in favor of the experimental (Schroth) group when compared to no intervention. The 95% confidence interval does not cross zero in the Kuru et al. study [11] indicating that we are 95% confident that the true mean between- group effect size for this study lies between .64 and 2.08. Alternatively, the effect size for the other study [26] does not yield the same confidence as indicated in Fig. 2.

The pooled data from the aforementioned 2 studies revealed significant heterogeneity by statistical testing (Cochrane Q = 6.33 *p* = .01; I^2^ = 84.2%). Therefore, no pooled effect sizes could be reliably reported for this group of studies.

### Does one PSSE method provide superior results compared to standard exercise?

**Fig. 3:**
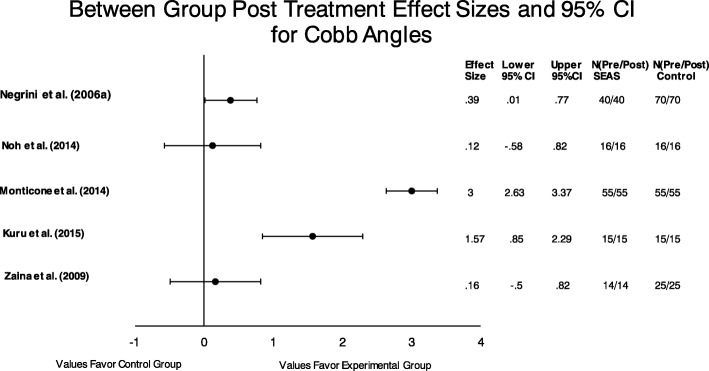
Between group effect sizes were calculated to answer the following question: Does one PSSE method provides superior results compared to standard exercise in patients with AIS? All studies included a SEAS experimental interventions except Kuru et al. used the Schroth method as the intervention group. Values to the right of the y axis indicate a positive effect, larger reduction in Cobb angles for the intervention group

Five between group effect sizes and their 95% confidence interval were calculated to answer the question (Fig. 3). All studies assessed under this sub-question compared the SEAS method, except Kuru et al. (2015), to a traditional form of exercise. A traditional form of exercise was defined by an in-house or home exercise intervention that was not specified to be a specific PSSE approach. Despite efforts to contact the authors, 2 studies [9, 28] effect sizes could not be calculated due to an incomplete data set. In general, effect sizes favored the experimental (SEAS) groups. The confidence intervals for Monticone et al. [27], Kuru et al. [11], and Negrini et al. 2006a [29] did not cross zero.

The pooled data from the aforementioned 5 studies revealed significant heterogeneity by statistical testing (Cochrane Q = 122.93 *p* < .00001; *I*^2^ = 96.75%). Therefore, no pooled effect sizes could be reliably reported for this group of studies.

## Discussion

The overall goal of this review was to determine if common PSEE exercise methods are more effective at improving Cobb angles compared to no intervention; to determine if a particular PSSE exercise method is more effective at reducing Cobb angles compared to standard exercise, and if possible, to compare one PSSE to another. It is important to keep in mind that all SEAS methods (e.g. SEAS, SEAS.02) were grouped together for the following comparisons, being collectively referred to as “SEAS”.

Overall, our results do not demonstrate compelling evidence that PSSEs are more effective at reducing Cobb angles when compared to observation or other modes of exercise. Unfortunately, there were no articles that directly compared one PSSE to another.

We were unable to pool data for the quantitative analysis for either question secondary to the lack of available studies and the heterogeneity of those studies that were available to analyze. In addition, the average quality of evidence for the studies included in this review was only moderate per the PEDro scale. These limitations alone make it difficult to make a strong conclusion either for or against the use of PSSEs compared to controls or other exercises.

More specific to our second question, the authors were able to collect several studies comparing the SEAS method to traditional exercise approaches. In general there were small to moderate effect sizes in favor of the SEAS approach. Two of the between group effect size confidence intervals crossed zero indicating that their remains some doubt as to whether the SEAS method is truly superior to traditional exercises. Therefore, based on the between group effect sizes and quality of these studies, the authors concluded that there is limited evidence that the SEAS approach is more effective than traditional exercises for the purpose of improving Cobb angles in patient with AIS.

Despite the paucity in the available evidence to answer our proposed questions, there are some trends in the data worth noting. All examined experimental groups demonstrated a positive effect for the reduction in Cobb angles indicating a beneficial trend in spine angle reduction (Figures 2 and 3). Two articles demonstrated very high effect sizes in favor of PSSE interventions, one Schroth and one SEAS [11, 27]. In addition, neither confidence interval crossed zero, so we can by 95% confident that the true mean effect would be positive for these particular populations. To that end, when looking at Figures 2 and 3 combined, four of the seven experimental groups did not cross zero [11, 27, 29].

Several difficulties were encountered when trying to effectively assess the effectiveness of PSSE approaches. A primary issue was that similar patient measurements were not consistently available in the reviewed manuscripts. Moreover, several articles lacked inclusion of both control and patient groups. A similar effect was seen between several Schroth articles meeting the initial search criteria. For example, only two of thirteen (~15.4%) documented both baseline and ending Cobb angles. An identical situation was noted for vital capacity values. These issues generated obstacles in defining an efficient method comparison mechanism. A comparable issue involving missing or incomplete bracing information was also noted. Incidentally, although accompanying brace application varied considerably it did not appear to have any noticeable impact on patient outcomes [28–31] when compared to studies without bracing [11, 25–27].

In assessing the PSSE articles during our original search, routinely employed comparable patient measurements and/or their corresponding results were difficult to find. The only consistent factor seen between these studies was that mean patient ages were generally between 12 and 15 years old. Substantial outcome measurement variation was exhibited in all other PSSE studies. Measured factors (Cobb angles, ATR, VC, SRS, and BME) were either not assessed or uniformly documented between all studies. This resulted in a noticeable lack of comparable information being documented in these remaining articles.Although currently considered a deficit, a lack of standardized PSSE outcome measurements also represents a unique opportunity to develop one. It is entirely feasible that a core set of measurements be established serving as a basis for performing predictive PSSE method comparisons. A primary requirement would be including both beginning and ending patient measurements along with associated statistical data (e.g. standard deviation). Additionally, a core measurement set does not have to be all that extensive to supply enough pertinent information. For example, it need only include three measurements (e.g. Cobb angles, ATR, VC) to provide relevant outcome information. Using such an approach does not necessarily mean that it has to be one of a restrictive nature. A flexible design would allow additional measurements (e.g. BME) to be added to the core set, which are deemed essential by clinicians to properly evaluate patient therapy progression. It should remain a top consideration when undertaking this process. The very nature of clinical practice dictates that PSSE provider retains flexibility in addressing individual patient needs. After all, he or she is ultimately responsible for determining the best indicators of success to remediate a patient’s abnormal spinal curvature(s).

Both Schroth and SEAS methods yielded positive outcomes despite differences in patient treatment modes. For instance, Schroth exercise method is primarily conducted on an outpatient basis in the presence of a clinician providing real time patient feedback. Alternatively, the SEAS method is first taught to the patient who then performs it on an at-home basis. The supervised, one-on-one approach of the Schroth method provides the added benefit of routinely ensuring that the patient is consistently performing the exercise correctly. This particular feature would have an inherent advantage over an SEAS method that relies mostly on unsupervised patient exercise. The patient may unintentionally; over time, deviate from the correct exercise approach. On the other hand, the outpatient approach used by the Schroth method may not be practical for all cases. Several factors including clinician availability, available transportation, treatment facility distance, and required equipment may limit PSSE choices. These factors and others should be taken into account when attempting to evaluate different PSSE methods.

There are limitations to this review when considering our collective findings. First, 2 papers included in this review could only be assessed qualitatively due to an incomplete data set. The author’s report in both studies that there were significant improvements in patient outcomes when comparing the PSSE group to traditional exercise groups, nevertheless the addition of two data points to Fig. 2 might substantially change our findings. Second, it is difficult to make general clinical recommendations about the findings of our review because of the aforementioned heterogeneity among the included studies. Third, we chose only to include articles written in English. Lastly, the results of this review are limited to objective structural measures (Cobb angles) and thus are not generalizable to self -reported patient outcomes.

## Conclusions

There is insufficient evidence to suggest that both Schroth and SEAS methods can effectively improve Cobb angles in patients with AIS compared to no intervention. There is limited evidence that the SEAS method is more effective at reducing Cobb angles compared to traditional exercises in treating AIS, while, there was no opportunity to compare one PSSE to another.

Because of the insufficient and limited evidence for PSSE efficacy, it is the authors’ opinion that patients with AIS would be better served if instituting a core set of measurements were considered. Such an endeavor could provide a more efficient PSSE evaluation mechanism. The ultimate goal of patient disease treatment is to achieve the best possible results using available resources. Consequently, clinicians should be provided the necessary means to make informed treatment determinations. Steps towards developing a uniform set of PSSE outcome measurements could help clinicians improve patient AIS treatment.

## Data Availability

Datasets used and/or analyzed during the current study are freely available from the corresponding author upon request.

## Appendix

### Studies Retrieved from the Initial Search

1. Dobosiewicz K, Durmala J, Czernicki K, Piotrowski J. Radiological results of Dobosiewicz method of three-dimensional treatment of progressive idiopathic scoliosis. Stud Health Technol Inform. 2006;123:267–72.
2. Negrini S, Negrini A, Romano M, Verzini N, Parzini S. A controlled prospective study on the efficacy of SEAS.02 exercises in preparation to bracing for idiopathic scoliosis. **Stud Health** Technol **Inform** 2006;519–522.
3. Negrini S, Negrini A, Romano M, Verzini N, Parzini S. A controlled prospective study on the efficacy of SEAS.02 exercises in preventing progression and bracing in mild idiopathic scoliosis. Stud Health Technol Inform. 2006;123:523–6.
4. Negrini S, Zaina F, Romano M, Negrini A, Parzini S. Specific exercises reduce brace prescription in adolescent idiopathic scoliosis: a prospective controlled cohort study with worst-case analysis. J Rehabil Med. 2008;40:451–5.
5. Negrini S, Negrini F, Fusco C, Zaina F. Idiopathic scoliosis patients with curves more than 45 Cobb degrees refusing surgery can be effectively treated through bracing with curve improvements. The Spinal Journal 2011;11:369–380.
6. Negrini S, Donzelli S, Lusini M, Zaina F. Bracing can reduce high degree curves and improve aesthetics immediately after the end of growth. Final results of a retrospective case series. Stud Health Technol Inform. 2012;176:393–6.
7. Kuru T, Yeldan I, Dereli EE., Ozdincler AR, Dikici F, Colak I. The efficacy of three-dimensional Schroth exercises in adolescent idiopathic scoliosis: a randomized controlled clinical trial. Clim Rehabil. 2016 Feb; 30(2): 181–190.
8. Otman S, Kose N, Yakut Y, 2005. The efficacy of Schroth’s 3-dimensional exercise therapy in the treatment of adolescent idiopathic scoliosis in Turkey. Saudi Med J 2005;26: 1429.
9. Schreiber S, Parent EC, Hedden, DM, Moreau M, Watkins EM. The effects of a 6-month Schroth intervention for Adolescent Idiopathic Scoliosis (AIS): preliminary analysis of an ongoing randomized controlled trial. *Scoliosis*. 2013;8(suppl 2): O44–5
10. Schreiber S, Parent EC, Hedden DM, Moreau M, Hill D, Lou E. Effects of Schroth exercises on curve characteristics and clinical outcomes in adolescent idiopathic scoliosis: protocol for a multicentre randomised controlled trial. Journal of Physiotherapy*.* 2014*;*60:234.
11. Schreiber S, Parent EC, Moez EK, Hedden DM, Hill D, Moreau MJ, Southon SC. The effect of Schroth exercises added to the standard of care on the quality of life and muscle endurance in adolescents with idiopathic scoliosis—an assessor and statistician blinded randomized controlled trial: “SOSORT 2015 Award Winner.” Scoliosis. 2015;10:1–12.
12. Moramarco M, Fadzan M, Moramarco K, Heller A, Righter S. The Influence of Short-Term Scolioisis-Specific Exercise Rehabilitation on Pulmonary Function in Patients with AIS. Current Pediatric Review. 2016;12:17–23.
13. Zaina F, Negrini S, Atanasio S, Fusco C, Romano M, Negrini A. Specific exercises performed in the period of brace weaning can avoid loss of correction in Adolescent Idiopathic Scoliosis (AIS) patients: Winner of SOSORT’s 2008 Award for Best Clinical Paper. Scoliosis*.* 2009;4:8.
14. Fabian KM, Rozek-Piechura K., 2014. Exercise tolerance and selected motor skills in young females with idiopathic scoliosis treated with different physiotherapeutic methods. Octop Traumatol Rehabil. 2014;16(5):507–22.
15. Fabian K. Evaluation of the effectiveness of asymmetric breathing exercises according to Dobosiewicz on chosen functional parameters of the respiratory system in girls with scoliosis. Fizjoterapia. 2010;18:21–26.
16. Monticone M, Ambrosini E, Cazaniga D, Rocca B, Ferrante S. Active self- correction and task-oriented exercises reduce spinal deformity and improve quality of life in subjects with mild adolescent idiopathic scoliosis. Results of a randomized controlled trial. Eur Spine J. 2014; 23(6): 1204–1214.
17. Weiss HR, Klein R. Improving excellence in scoliosis rehabilitation: A con- trolled study of matched pairs. Pediatr Rehabil. 2006; 9(3):190–200.
18. Parent EC, Schrieber S, Hedden DM, Moreau M, Hill D, Watkins E. The effect of a 6-month Schroth exercise program: a pilot study using subjects as their own controls. Scoliosis. 2013;8(Suppl 2):O45–6.
19. Noh DK, You JS, Koh JH, Kim H, Kim D, Ko S-M, Shin J-Y. Effects of novel corrective spinal technique on adolescent idiopathic scoliosis as assessed by radiographic imaging. Journal of Back and Musculoskeletal Rehabilitation*.* 2014;27:331–338.
20. Borysov M, Mogiliantseva T. Rehabilitation of Adolescents with Scoliosis During Growth - Preliminary Results Using a Novel Standardized Approach in Russia. (Methodology). Curr Pediatr Rev. 2016;12(1):31–5.
21. Borysov M, Borysov A. Scoliosis short-term rehabilitation (SSTR) according to ‘Best Practice’ standards are the results repeatable? Scoliosis. 2012; 7: 1.
22. Pugacheva N. Corrective exercises in multimodality therapy of idiopathic scolio- sis in children - analysis of six weeks efficiency-pilot study. Stud Health Technol Inform. 2012;176:365–71.
23. W. A. den Boer P. G. Anderson J. v. Limbeek M. A. P. Kooijman. Treatment of idiopathic scoliosis with side-shift therapy: an initial comparison with a brace treatment historical cohort. Eur Spine J (1999) 8:406–410.
24. Schreiber S. et al. Schroth Physiotherapeutic Scoliosis-Specific Exercises Added to the Standard of Care Lead to Better Cobb Angle Outcomes in Adolescents with Idiopathic Scoliosis - an Assessor and Statistician Blinded Randomized Controlled Trial. PLoS One. 2016; 11(12): e0168746.

## Abbreviations

AIS: Adolescent idiopathic scoliosis
ATR: Axial trunk rotation
BME: Biering-Sorensen endurance test
CI: Confidence interval
CINAHL: Cumulative Index of Nursing and Allied Health Literature
CTLSO: Cervico-thoraco-lumbo-sacral orthosis
DoboMed: Dobosiewicz method
PEDro: Physiotherapy Evidence Database
PSSE: Physiotherapy scoliosis specific exercise
SEAS: Scientific Exercise Approach to Scoliosis
SRS: Scoliosis Research Society
TLSO: Thoracolumbar-sacral orthosis
VC: Vital Capacity
